# Supplementary material for: A Global Screen for Assembly State Changes of the Mitotic Proteome by SEC-SWATH-MS
Source: Cell Syst. 2020 Feb 26;10(2):133–155.e6. doi: 10.1016/j.cels.2020.01.001 (PMC7042714; doi:10.1016/j.cels.2020.01.001)

Q9UEU0 | VTI1B_HUMAN | VTI1B VTI1 VTI1L VTI1L1 VTI2
Monomer MW [kDa]: 26.688 Monomer expected elution fraction: 52

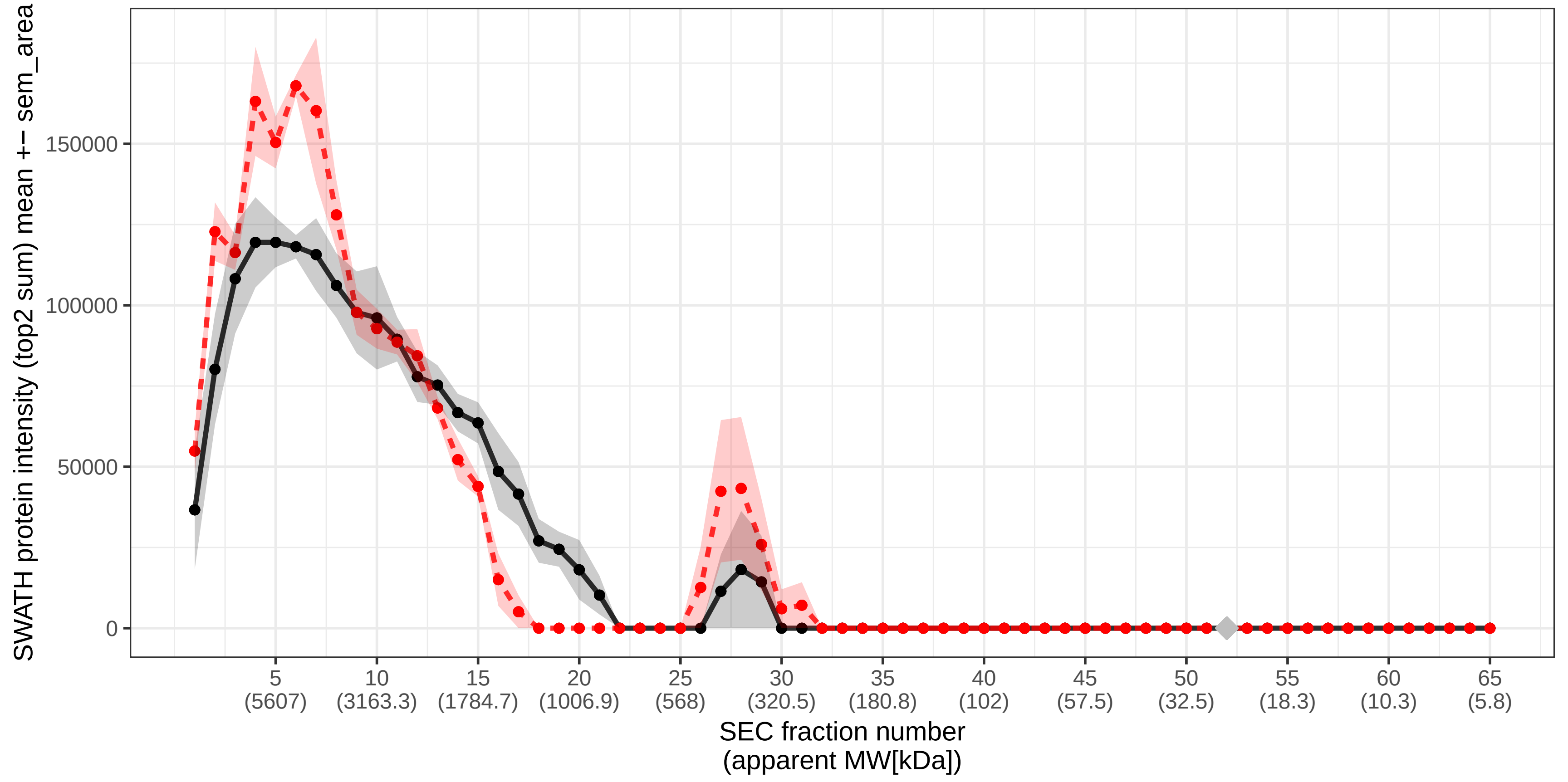

Supplement: Data S1. SEC-SWATH-MS Protein Chromatograms, Related to Figure 1 [file mmc6.zip › SECchrom_Q9UEU0_VTI1B_HUMAN_VTI1B_VTI1_VTI1L_VTI1L1_VTI2.pdf]
